# Effect of Intravenous Sodium Valproate vs Dexamethasone on Acute Migraine Headache: A Double Blind Randomized Clinical Trial

**DOI:** 10.1371/journal.pone.0120229

**Published:** 2015-03-20

**Authors:** Shahir Mazaheri, Jalal Poorolajal, Akram Hosseinzadeh, Mohammad Mahdi Fazlian

**Affiliations:** 1 Department of Neurology, School of Medicine, Hamadan University of Medical Sciences, Hamadan, Iran; 2 Modeling of Noncommunicable Diseases Research Center and Department of Epidemiology & Biostatistics, School of Public Health, Hamadan University of Medical Sciences, Hamadan, Iran; 3 Hamadan District Health Center, Hamadan University of Medical Sciences, Hamadan, Iran; 4 Beasat Hospital, Hamadan University of Medical Sciences, Hamadan, Iran; University of Ottawa, CANADA

## Abstract

**Background:**

Despite the impact of sodium valproate and dexamethasone on migraine headache, the efficacy of the two drugs has not been properly investigated and compared. This trial compared the effect of the two drugs on acute migraine headache.

**Methods:**

This double blind randomized clinical trial was conducted on patients aged 18 to 65 years with acute migraine headache who referred to the emergency departments of Beasat and Farshchian Hospitals in Hamadan, Iran, from April 2012 to June 2014. Patients were randomly assigned to receive a single-dose of either 400 mg sodium valproate or 16 mg dexamethasone plus 50 ml saline normal solution within 15 min intravenously. The severity of headache in the two groups was evaluated at baseline, 0.5 and 2 hours later using the Visual Analog Scale (VAS) on a scale of 0 to 10.

**Results:**

Of 104 patients enrolled, 72 patients remained for analysis. The effect of both sodium valproate and dexamethasone on acute migraine headache was statistically significant at 0.5 and 2 hours post-treatment compared to pre-treatment (P=0.001). The severity of headache based on VAS reduced form 8.20 (7.72, 8.68) before treatment to 5.31 (4.74, 5.89) and 3.66 (2.99, 4.33) at 0.5 and 2 hours after treatment, respectively, in patients receiving sodium valproate and from 8.46 (8.05, 8.86) before treatment to 5.46 (4.81, 6.11) and 3.59 (2.84, 4.35) at 0.5 and 2 hours after treatment, respectively, in patients receiving dexamethasone. Both drugs were highly effective in improvement of acute headache in patients without aura. However, sodium valproate significantly improved the acute headache in patients with aura but dexamethasone did not. The severity of headache based on VAS reduced form 8.50 (7.40, 9.60) before treatment to 4.67 (2.40, 6.93) and 3.50 (1.78, 5.22) at 0.5 and 2 hours after treatment, respectively, in patients with aura receiving sodium valproate and from 8.80 (7.76, 9.84) before treatment to 7.20 (4.98, 9.42) and 6.20 (2.43, 9.97) at 0.5 and 2 hours after treatment, respectively, in patients with aura receiving dexamethasone.

**Conclusions:**

This trial indicated that, in overall, intravenous sodium valproate is not superior to intravenous dexamethasone in treatment of acute migraine attacks. However, in patients with aura, only sodium valproate but not dexamethasone is effective in headache relief. This issue needs further investigations.

**Trial Registration:**

ClinicalTrials.gov IRCT201202199014N1

## Introduction

Migraine is the most frequent cause of emergency department visits for headache [1,2]. Several parenteral medications are used to treat acute migraine, but published data indicate that none offer rapid and complete headache relief without side effects [3]. Medications used for acute migraine headache target the serotonergic system, the inflammatory reaction or calcitonin gene-related peptide receptors [4].

Several case series [5–8] and few randomized clinical trials [9] have investigated the safety and efficacy of intravenous sodium valproate in acute migraine attacks. Furthermore, many published reports suggested that dexamethasone is significantly effective in acute migraine [10,11]. Nonetheless, the results of current clinical trials addressing the efficacy of sodium valproate and dexamethasone are inconsistence. Some trials reported that both drugs are effective in managing acute migraine attacks [11–15] while the others reported opposite results [16–19].

Despite the impact of sodium valproate and dexamethasone on migraine headache, the efficacy of the two drugs has not been properly investigated and compared. The only information comes from small, unrepresentative randomized clinical trials [9]. Until reliable information on the safety and efficacy of the two drugs is collected in different settings, it is difficult to make an effective clinical decision on management of emergency conditions associated with migraine attacks. This clinical trial was designed to assess and compare the benefits and harms of intravenous (IV) sodium valproate and dexamethasone on acute migraine headache in patients who referred to emergency departments.

## Materials & Methods

The protocol for this trial and supporting CONSORT checklist are available as supporting information; see S1 Checklist and S1 and S2 Protocols. The protocol was registered with the Iranian Registry of Clinical Trials on March 24, 2012 (IRCT201202199014N1). The protocol was updated for changing the dose of dexamethasone from 8 mg to 16 mg.

This double blind randomized clinical trial was conducted at Beasat and Farshchian Hospitals, affiliated with Hamadan University of Medical Sciences, the west of Iran, from April 2012 to June 2014. Written informed consent was received from all patients. The participants were recruited and followed from April 2012 to March 2014. The 'Regional Ethics Committee' of Hamadan University of Medical Sciences approved the consent procedure and the whole trial.

According to the results of previous clinical trials, the proportion of migraine headache improved by sodium valproate and dexamethasone was 73% [6] and 33% [10], respectively. On the basis of these results, we arrived at a sample size of 36 for each group and a total sample size of 72 at 95% significance level (the type I error) and 90% statistical power (the type II error), considering a two-sided hypothesis. One-half of the difference between the two drugs, i.e., 20%, was determined for the clinically significant level to support the conclusion.

The study population included patients aged 18 to 65 years with a history of having migraine for at least one year, who referred to emergency departments of Beasat or Farshchian hospitals for an acute migraine headache with severity equal to or greater than 5 score based on the Visual Analog Scale (VAS), on a scale of 0 to 10. Zero denoted no pain, and 10 denoted the most severe pain that the patient had ever experienced (pain plus crying). Migraine was defined according to the International Classification of Headache disorders, 2nd edition [20].

Patients with a history of migraine, regardless of the type of migraine (e.g., chronic or cluster), who referred to the emergency departments because of acute headache were enrolled into the study. However, patients with any of the following characteristics were excluded from the study: (a) hypersensitivity to sodium valproate or dexamethasone; (b) systolic blood pressure less than 100 or greater than 140 mm Hg; (c) heart rate less than 65 bit per min; (d) episode of tension headache during the last month; (e) using analgesic, ergotamine, or oral sodium valproate within eight hours before the time of enrollment; (f) known systemic diseases such as diabetes, peptic ulcer, liver disease, renal failure, malignancy, or epilepsy; and (g) acute infection or inflammatory disease at the time of enrollment.

The eligible patients were randomly assigned to two groups using the balance block randomization method as follows. We prepared 4 sheets of paper, writing on 2 sheets “V” for “valproate” and on 2 “D” for “dexamethasone”. The paper sheets were pooled, placed in a container, and randomly drawn one at a time for each patient without replacement until all 4 sheets were drawn. The 4 paper sheets were then placed back into the container and this action repeated until the sample size was reached. The allocations remained concealed and the nature of medications remained blind during the study so that neither the examiner nor the patients knew (were blind to) the nature of the medications. For this purpose, the drugs were already put in envelopes labeled a serial number from 1 to 72. No one knew the nature of the envelopes except the coordinator of the trial (SM).

The intervention group received a single dose of 400 mg sodium valproate plus 50 ml saline normal solution within 15 min intravenously and the control group received a single dose of 16 mg dexamethasone plus 50 ml saline normal solution within 15 min intravenously.

The primary outcomes of interest were measuring the severity of headache using VAS scale as well as presence or absence of nausea and photophobia which were evaluated at baseline, 0.5 and 2 hours later. The secondary outcomes of interest were episodes of any side effects such as nausea, sedation, and face paresthesia which were evaluated at baseline and 2 hours after intervention.

The independent and paired t-tests were used and repeated measure ANOVA for analyzing continuous variables and chi-square test for analyzing categorical variables. All statistical analyses were performed at a significance level of 0.05 using Stata software, version 11 (StataCorp, College Station, TX, USA).

## Results

**Fig 1:**
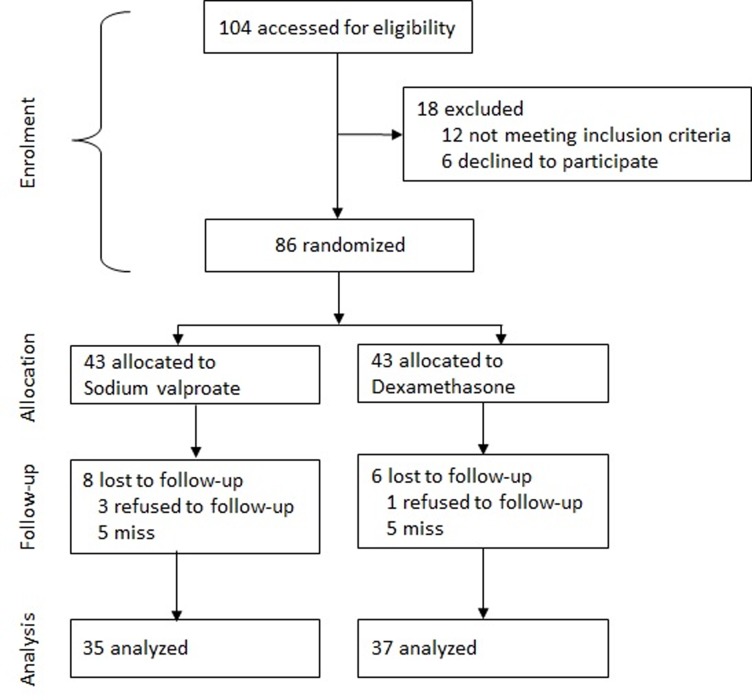
Flowchart of progress through the trial.

**Fig 2:**
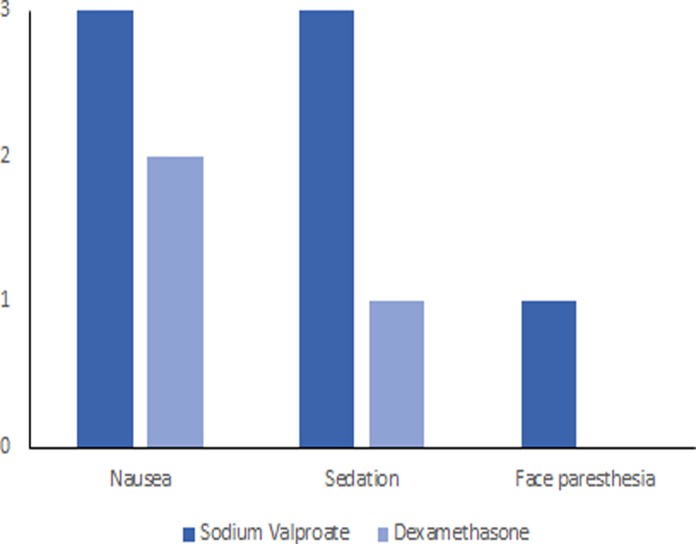
Comparison of the treatment side effects among sodium valproate and dexamethasone groups 2 hours post-treatment.

Of 104 patients identified, 12 were ineligible and 6 declined to participate. The randomization was based on the remaining 86 patients, of whom 43 patients were allocated to the intervention (sodium valproate) group and 43 to the control (dexamethasone) group. Fourteen patients declined follow-up including 8 patients in the intervention group and 6 in the control group. The analysis was based on data from the remaining 72 patients (13 men and 59 women) including 35 in the intervention group and 37 in the control group (Fig. 1). The mean (SD) age of the patients was 34.6 (10.7) years. The characteristics of the participants in the intervention (sodium valproate) and control (dexamethasone) groups are given in Table 1. No serious adverse effects were noted in the two groups. However, minor adverse effects including nausea, sedation, and face paresthesia were reported by a few patients (Fig. 2).

**Table 1:** Comparison of the characteristics of the study population by the type of drugs administered using chi-square test.

Variables	Sodium Valproate n = 35	Dexamethasone n = 37	*P* value
**Age** (**yr**)			0.036
Mean age (SD)	37.29 (11.7)	32.05 (9.1)	
**Sex** (%)			0.845
Male	6 (17.1)	7 (18.9)	
Female	29 (82.9)	30 (81.1)	
**Aura** (%)			0.669
No	29 (82.9)	32 (86.5)	
Yes	6 (17.1)	5 (13.5)	
**Menstruation period** (%) ^a^			0.708
No	8 (27.6)	7 (23.3)	
Yes	21 (72.4)	23 (76.7)	
**Nausea at baseline**			0.224
No	7 (20.0)	4 (10.8)	
Yes	28 (80.0)	33 (89.2)	
**Photophobia at baseline**			0.925
No	5 (14.3)	5 (13.5)	
Yes	30 (85.7)	32 (86.5)	
**Recovered from nausea** ^b^			0.176
No	6 (21.4)	3 (9.1)	
Yes	22 (78.6)	30 (90.9)	

^a^ in women

^b^ Only patients with nausea at baseline were evaluated

The effect of sodium valproate versus dexamethasone on migraine headache are given in Table 2. There was no statistically significant difference in the severity of headache between the two groups on pre-treatment (*P* = 0.405) and 0.5 (*P* = 0.736) and 2 (*P* = 0.901) hours post-treatment. In other words, the two drugs had the same effect on the improvement of migraine headache. We performed repeated measures ANOVA and found no statistically significant difference between the effects of two drugs on acute migraine headache (*P* = 0.764).

**Table 2:** The effect of sodium valproate (n = 35) compared to dexamethasone (n = 37) on acute migraine headache, based on the VAS mean score of 1 to 10, using univariate (t-test) and repeated measures ANOVA analysis.

	Mean score of headache (95% CI)		t-test	Repeated Measure ANOVA
Treatment	Sodium valproate	Dexamethasone	P value	P value
Pre-treatment	8.20 (7.72, 8.68)	8.46 (8.05, 8.86)	0.405	0.764
Post-treatment 0.5 hr	5.31 (4.74, 5.89)	5.46 (4.81, 6.11)	0.736	
Post-treatment 2 hr	3.66 (2.99, 4.33)	3.59 (2.84, 4.35)	0.901	

The effect of sodium valproate and dexamethasone on acute migraine headache pre-treatment compared to post-treatment are given in Table 3. The effect of both drugs on acute migraine headache was statistically significant at 0.5 and 2 hours post-treatment compared to pre-treatment (*P* = 0.001). In addition, both drugs were highly effective in improvement of acute headache in patients without aura. However, the results were completely different in patients with aura. As shown in the table, sodium valproate significantly improved the acute headache in patients with aura (*P* = 0.001) but dexamethasone did not (*P* = 0.166). In addition, the effect of sodium valproate and dexamethasone on pain relief were compared in patients with and without aura separately. As shown in Table 3, the effect of the two drugs on pain relief was the same in patients without aura. However, the effect of sodium valproate was significantly higher than that of dexamethasone on pain relief in patients with aura.

**Table 3:** The effect of sodium valproate (n = 35) and dexamethasone (n = 37) on acute migraine headache pre-treatment compared to 0.5 and 2 hours post-treatment, based on the VAS mean score of 1 to 10, using a t-test.

	Pre-treatment	Post-treatment 0.5 hr		Post-treatment 2 hr	
Stage	Mean (95% CI)	Mean (95% CI)	P value	Mean (95% CI)	P value
**Total patients**					
Sodium Valproate	8.20 (7.72, 8.68)	5.31 (4.74, 5.89)	0.001	3.66 (2.99, 4.33)	0.001
Dexamethasone	8.46 (8.05, 8.86)	5.46 (4.81, 6.11)	0.001	3.59 (2.84, 4.35)	0.001
P value	0.405	0.736		0.901	
**Patients with aura**					
Sodium Valproate	8.50 (7.40, 9.60)	4.67 (2.40, 6.93)	0.001	3.50 (1.78, 5.22)	0.001
Dexamethasone	8.80 (7.76, 9.84)	7.20 (4.98, 9.42)	0.160	6.20 (2.43, 9.97)	0.166
P value	0.184	0.001		0.001	
**Patients without aura**					
Sodium Valproate	8.14 (7.57, 8.70)	5.45 (4.85, 6.05)	0.001	3.69 (2.91, 4.46)	0.001
Dexamethasone	8.41 (7.95, 8.86)	5.19 (4.52, 5.85)	0.001	3.19 (2.52, 3.86)	0.001
P value	0.408	0.522		0.281	

**Fig 3:**
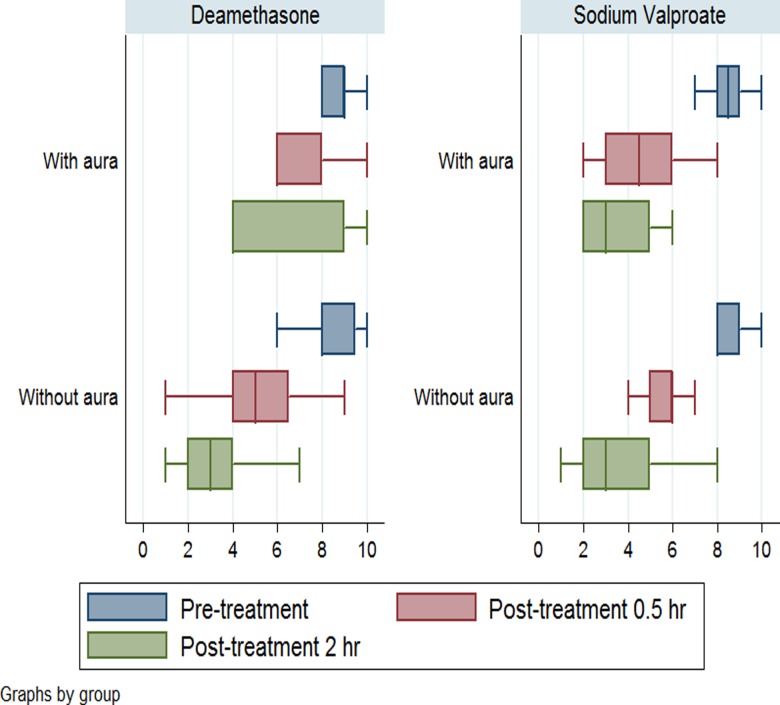
Box plots based on the VAS mean score of 1 to 10 for migraine headache across intervention groups including sodium valproate (n = 35) and dexamethasone (n = 37)

Box plots based on the VAS mean score of 1 to 10 for migraine headache across intervention groups including sodium valproate and dexamethasone are given in Fig. 3. The results indicates that the the effects of the two drugs were nearly the same in pateints withour aura but not in patients with aura. In patients with aura, sodium valproate was more effective than dexamethasone in pain relief.

## Discussion

Current studies have revealed that both sodium valproate [5–8] and dexamethasone [2,9], can be effectively used for treatment of migraine headache. We indicated that both sodium valproate and dexamethasone had substantial effects on migraine attack. However, the patients with aura responded well to sodium valproate but not to dexamethasone, although patients without aura responded well to the both drugs. This issue may be related to the pathophysiologic mechanisms of migraine with aura. Migraine attacks with auras are thought to be associated with underlying hereditary or acquired neurovascular disorders [21]. Abnormal cortical excitability has been suggested to play an important role in the pathogenesis of migraine. Particularly, a failure of inhibitory circuits in migraine with aura been suggested. Sodium valproate acts as a central GABA and is able to modify cortical excitability state and improve the intracortical circuits in migraine [22].

Consistent with our findings, the results from a randomized clinical trial showed no significant difference between the therapeutic effect of IV sodium valproate and dexamethasone on migraine headache [9]. However, the results of current clinical trials comparing the effect of sodium valproate or dexamethasone with other medications are inconsistence. Bakhshayesh et al [13] conducted a clinical trial to compare the effect of IV sodium valproate with intramuscular metoclopramide plus subcutaneous sumatriptan on acute migraine attacks and reported that IV sodium valproate was more effective than metoclopramide plus sumatriptan while another clinical trial conducted by Friedman et al [12] revealed that IV valproate was inferior to metoclopramide or ketorolac in improving headache outcomes in patients with acute migraine attack. In addition, Fiesseler et al [16] performed a double-blind placebo-controlled randomized trial and indicated that steroids had no significant effect on the recurrence of migraine headaches while another double-blind placebo-controlled randomized multicenter trial conducted by Friedman et al [11] reported a 38% pain-relief in patients with acute migraine headache who were treated with dexamethasone. The evidence comes from clinical trials conducted in different settings suggests that no global consensus is there on the efficacy and implication of IV sodium valproate and dexamethasone in management of acute migraine attacks.

The main limitation of the present study was the small number of migraine patients with aura (11 out of 72 patients). The reason was that we knew nothing about the interaction between the aura and the efficacy of the two drugs. There was no evidence about this issue in the current literatures either. Therefore, we did not consider this (patients with and without aura) during the sampling. Although the subgroup of migraine patients with aura was small, the results of this subgroup were statistically significant. However, the results come from a small subgroup. This issue may raise the possibility of random error and needs further investigations in the future studies. Another limitation of this study was that the efficacy of the two drugs was examined for a short period of time. We did not evaluate the long-term effects of the two drugs on the relapse of migraine attacks. This issue needs to be investigated by designing long-term randomized clinical trials. Despite its limitations, this trial was able to properly assess and compare the efficacy of IV sodium valproate and dexamethasone in the management of patients with acute migraine attacks who seek emergency intervention for headache relief.

## Conclusion

This trial indicated that IV sodium valproate was not superior to IV dexamethasone in the treatment of acute migraine headache. But, in patients with aura, sodium valproate effectively improved migraine headache but dexamethasone did not. However, more evidence based on large randomized clinical trials is needed to assess the effectiveness of the two drugs on migraine headache with aura.

## Supporting Information

S1 Checklist CONSORT Checklist. (PDF)

S1 Protocol Protocol English. (DOCX)

S2 Protocol Protocol Persian. (DOC)
