# Supplementary material for: Effect of Intravenous Sodium Valproate vs Dexamethasone on Acute Migraine Headache: A Double Blind Randomized Clinical Trial
Source: PLoS One. 2015 Mar 20;10(3):e0120229. doi: 10.1371/journal.pone.0120229 (PMC4368536; doi:10.1371/journal.pone.0120229)
Supplement: S2 Protocol — (DOC) [file pone.0120229.s003.doc]

**فرم پيشنهاد طرح پژوهشي**

*عنوان طرح:*

بررسی اثر سدیم والپروات وریدی نسبت به دگزامتازون بر سردرد حاد میگرنی: كارآزمايي باليني تصادفي شده دو سو كور

*نام و نام خانوادگي مجري طزح:*

دکتر شهیر مظاهری

*دانشكده / گروه پژوهشي:*

دانشکده پزشکی / گروه نورولوژی

# قسمت اول - اطلاعات مربوط به عوامل اجرايي طرح

**توجه : چنانچه طرح ،دانشجويي يا پايان نامه مي‌باشد لازم است اين صفحه به طور جداگانه براي دانشجو و استاد راهنما تكميل شود.**

1. نام و نام خانوادگي طرح دهنده : **دکتر شهیر مظاهری**
2. رتبه علمي : استادیار
3. محل خدمت : بیمارستان فرشچیان
4. نشاني محل خدمت : همدان خیابان میرزاده عشقی بیمارستان فرشچیان
5. تلفن محل خدمت : 8285011
6. نشاني پست الكترونيك : dr_sh_mazaheri@yahoo.com
7. نشاني يا تلفن براي دسترسي سريع و پيامهاي فوري : 09181116858
8. درجات علمي و سوابق تحصيلي طرح دهنده به ترتيب از ليسانس به بعد ذكر گردد

| سال دريافت | كشور | دانشگاه يا محل تحصيل | رشته تحصيلي وتخصصي | درجه تحصيلي |
| --- | --- | --- | --- | --- |
| 1368 | ایران | اهواز | پزشک عمومی | دکتری |
| 1373 | ایران | اصفهان | نورولوژی | تخصص |

1. آيا تا كنون دوره هاي روش تحقيق را گذرانده ايد ؟ بلي × خير  در صورت پاسخ مثبت، جدول زير را تكميل فرماييد .

| **محل برگزاري دوره** | **تاريخ** | **سطح دوره ونوع آموزشهاي ارائه شده** |
| --- | --- | --- |
|  |  | مقدماتی و پیشرفته |

1. مشخصات همكاران اصلي^3^ طرح :

| امضاي همكار | نوع همكاري | درجه علمي | شغل | نام و نام خانوادگي | رديف |
| --- | --- | --- | --- | --- | --- |
|  | آنالیز آماری اطلاعات و مشاوره آماری | استادیار | استادیار اپیدمیولوژی | **دکتر جلال پورالعجل** | 2 |
|  | نظارت و تجویز دارو | دکترا | پزشک عمومی | **دکتر مهدی فضلیان** | 3 |
|  | نظارت و تجویز دارو | دکترا | پزشک عمومی | ----- | 4 |
|  | تزریق دارو | لیسانس | کارشناس پرستاری | **----** | 5 |
|  | جمع آوری اطلاعات | فوق لیسانس | کارشناس | **حمید اخوان** | 6 |

**قسمت دوم – اطلاعات مربوط به طرح پژوهشي**

**عنوان طرح به فارسي**

بررسی اثر سدیم والپروات وریدی نسبت به دگزامتازون بر سردرد حاد میگرنی: كارآزمايي باليني تصادفي شده دو سو كور

**عنوان طرح به انگليسي**

The Effect of Intravenous Sodium Valprate versus Dexamethason on Acute Migrane Headache: A Double Blinded Randomized Clinical Trial

**نوع طرح**

**كاربردي** بنيادي بنيادي-كاربردي جامعه نگر(HSR)

**بیان مسئله**

میگرن تقریبا 12% جمعیت در اغلب کشورهای غربی را گرفتار کرده است. سازمان بهداشت جهانی میگرن را به عنوان یکی از 20 بیماری ناتوان کننده جهان محسوب کرده است. اهمیت آن از نظر بیماری و هزینه بیشتر از مجموع بیماریهای صرع، استروک، سندرم پارکینسون، MS و آلزایمر می‌باشد . بر اساس یک آمار، میگرنی ها به طور میانگین به 6/5 (زنان) و 8/3 (مردان) روز استراحت در این شرایط نیاز دارند و هزینه مستقیم پزشکی آنها در سال 1 میلیارد دلار تخمین زده شده است .

طبق ملاکهای تشخیصی طبقه بندی دوم انجمن بین المللی سردرد (IHS ) در سال 2004 میلادی میگرن به دو نوع میگرن با اورا و بدون اورا تقسیم بندی می‌شوند. درمان بایستی با داروهایی که بالاترین سطح تاثیر و کمترین عوارض جانبی دارند شروع گردند. درمان غیرداروئی بیماران شامل اجتناب از تحریک کننده ها،خواب منظم، رژیم و فعالیت مناسب و دوری از استرس می‌باشد. درمان داروئی شامل درمان پروفیلاکسی و درمان حاد می‌باشد. درمان حاد شامل درمان میگرن به طور اولیه با داروهای رایج برای بهبودی سریع و مداوم درد بدون عود آن در 24 ساعت و ترجیحا در 48 ساعت اول می‌باشد. درمان حاد میگرن شامل مسکن ها (مخدر و غیر مخدر)، آلکالوئیدهای ارگو، تریپتان ها و کورتیکواستروئیدها میباشند . مسکن ها صرفا در موارد خفیف بدون تهوع و داروهای گروه دوم وسوم در موارد فشارخون بالا، بیماریهای قلبی و حاملگی موارد منع استعمال دارند. بعضی مطالعات استفاده از استروئید iv در استاتوس میگرن و سندرم های overuse را توصیه می نماید ولی به خاطر عوارضی مانند هیپرگلیسمی، زخم های گوارشی، گلوکوم و غیره همه جا کاربرد ندارد . هدف درمان پیشگیری بهبود کیفیت زندگی با کاهش فرکانس، شدت و مدت حملات و همچنین با افزایش پاسخ میگرن حاد به درمان می باشد .

مکانیسم میگرن در حال حاضر به صورت کمپلکسی از پروسه های مختلفی مانند تغییرات Input های درد و حس ، افزایش حساسیت کورتکس به فنومن اورا ، تسهیل مرکزی درد ، التهاب نروژنیک و حساسیت گیرنده های درد در ساقه مغز مشخص می شود . آن عمدتاً دیاسفال و ساقه مغز را تحت تاثیر قرار می دهد و مثالی از تقویت غیرطبیعی و حساسیت راه های درد در این نواحی محسوب می شود . در مطالعه Pappagallo در سال 2004 مشخص شده است که داروهای ضد تشنج مثل سدیم والپروات و توپیرامات ممکن است با مهار کانال های سدیم ، مهار کانال های کلسیمی یا با مهار GABA اثرات سودمندی در بیماران میگرنی داشته باشد .

والپرات خوراکی در پروئیداکسی میگرن موثر است . والپروات دو مکانیسم عمل مرکزی و محیطی ( بیرون مغز ) دارد . والپروات با چندین مکانیسم روی سیستم عصبی مرکزی تاثیر می گذارد . اول والپروات سطح GABA را در مغز با فعالیت آنزیم گلوتامیک اسید دکربوکسیداز و مهار GABA آمینوترانسفراز و سوکسینات سمی آلدئید دهیدروژناز افزایش می دهد ( آنزیم تجزیه کننده GABA ) . دوم : والپروات تحریک سلولهای سروتونرژیک در دورسال رافه را که در سردرد نقش دارد . سوم: والپروات فعالیت مرکزی تری ژمینال را کاهش میدهد. اثر محیطی والپروات (خارج از سیستم عصبی مرکزی )کاهش التهاب نروژنیک آزمایشگاهی در سیستم عروق تری ژمینال که از طریق آگونیست ژیتور GABA اعمال اثر می کند .^14^در بخش اورژانس درمان میگرن حاد اغلب به صورت تزریق است . این به علت بی اثری داروهای خوراکی به علت تهوع و استفراغ و یا عدم توانایی در تحمل تریپتان ها یا صلاحدید پزشک و یا خواسته بیمار برای بهبود سریع درد باشد . در عین حال نتایج متاآنالیز جدید اثرات تجویز سدیم والپروات را در حمله حاد میگرن مشخص نشده است. با توجه به اینکه تا کنون مطالعه کارآزمایی بالینی با هدف مقایسه اثر درمانی سدیم والپروات و دگزامتازون بر درمان حاد سردرد میگزنی صورت نگرفته است و مطالعات پیشین ضرورت انجام چنینی مطالعه‌ای را توصیه نموده‌اند مطالعه حاضر با هدف بررسی اثر و safety سدیم والپروات وریدی (دپاکن) و دگزامتازون را در درمان میگرن حاد به صورت دو سوکور تصادفی و آینده نگر طراحی شده است.

**سابقه بررسی متون**

درمطالعه Mathew و همکاران در سال 2000 با تزریق وریدی والپروات سدیم کاهش بیش از 50% در شدت سردرد در نیم ساعت اول در 48 از 66 (73%) مشاهده شد و هیچ عوارض جانبی گزارش نشد .

در مطالعه Tanen و همکاران به صورت دو سوکور و تصادفی در سال 2003 از نظر آماری و بالینی پروکلرپرازین بر سدیم والپروات در درمان میگرن حاد ارجح شناخته شد .

در مطالعه دیگر به روش غیر تصادفی و بدون کورسازی 20 بیمار با حملات مکرر غیر کنترل شده 4 میلی گرم دگزامتازون همراه با رژیم غیر استاندارد تریپتان ها و NSAID برای کاهش عود سردرد به کار برده شد و مشخص شد که اضافه شدن دگزامتازون عود میگرن را کاهش می‌دهد .

در مطالعه‌ای که توسط Stilman و همکاران بر روی 130 بیمار میگرنی اجام شد نشان داد که تجویر سدیم والپروات به تنهایی باعث درمان سریع و ایمن حملات حاد میگرن شده و عارضه جانبی نداشته است و نقش یک مسکن را در درمان سردرد حاد میگرنی بازی می‌کند. در این مطالعه کارزآمایی بالینی برای برری اثر این دارو در بیماران مبتلا به سردرد حادی میگرنی توصیه شده است .

در مطالعه دیگری که توسط Shahien بر روی بیماران مبتلا به سردرد حاد میگرنی انجام دادند نشان دادند که داروی سدیم والپروات می‌تواند به طور ایمنی و سریع سردرد حاد میگرنی را تسکین دهد. در این مطالعه به انجام کارآزمایی بالینی توصیه شده است .

مطالعه‌ی موردی که توسط Avraham و همکاران بر روی دختر 12 ساله مبتلا به حمله حاد میگرنی شده بود نشان داد که تزریق سدیم والپروات توانست به صورت سریع و مؤثر سردرد بیمار را تسکین دهد .

**اهداف اصلي طرح**

تعيين اثر دگزامتازون و سديم والپروات وريدي بر حمله میگرن حاد

**اهداف فرعي طرح**

1. تعيين اثر والپروات سدیم وریدی بر حمله حاد میگرن
2. تعيين ميزان بروز عوارض جانبی سديم والپروات وریدی
3. تعيين اثر دگزامتازون وریدی بر حمله حاد میگرن
4. تعيين ميزان بروز عوارض جانبي دگزامتازون وریدی
5. تعيين اندازه اثر سديم والپروات سدیم وریدی (دپاکن) نسبت به دگزامتازون وريدي بر حمله حاد میگرن

**هدف كاربردي طرح**

چنانچه اثر سديم والپروات وريدي بر حمله حاد ميگرن بيشتر و اثرات جانبي آن كمتر از دگزامتازون وريدي باشد مي‌توان اين دارو را به عنوان داروي خط اول درمان ميگرن حاد پيشنهاد نمود.

**سؤالات پژوهش**

1. اثر والپروات سدیم وریدی بر حمله حاد میگرن چقدر است.
2. ميزان بروز عوارض جانبی سديم والپروات وریدی چقدر است؟
3. اثر دگزامتازون وریدی بر حمله حاد میگرن چقدر است؟
4. ميزان بروز عوارض جانبي دگزامتازون وریدی چقدر است؟

**فرضیات پژوهش**

اثر سديم والپروات وريدي بر حمله حاد ميگرن نسبت به دگزامتازون وريدي متفاوت است.

**نوع مطالعه**

مطالعه كارآزمايي باليني تصادفي شده دو سو كور (Double blinded randomized clinical trial)

**روش اجرا**

بیماران بالغ که با حمله حاد سردرد میگرنی (با سابقه حداقل یک سال) که به بخش اورژانس بیمارستان‌های دانشگاهي فرشچیان و بعثت مراجعه می‌کنند در صورت داشتن رضايت وارد مطالعه خواهند شد.. تشخیص براساس طبقه‌بندی بین‌المللی اختلالات سردرد (IHS ) ملاک انجمن بین‌المللی سردرد میباشد .^3^ که بعد از معاینه و تشخیص توسط مجری طرح به صورت یک روز در هفته در درمانگاه نرولوژی مورد بررسی قرار می‌گیرد و در صورت مطابقت وارد طرح میگردند.

معیارهای ورود

1. سن 18 تا 65 سال.
2. داشتن سابقه ميگرن طبق ملاک IHS برای بیش از یک سال.
3. شدت درد بیش از 5 با Visual analogue scale (VAS )

معیارهای خروج

1. فشار خون سیتولیک زیر 100 ميلي‌متر جيوه و یا ابتلا به فشارخون بالای 140 روي 90
2. ضربان قلب زیر 65 در دقیقه
3. سابقه ابتلا به بیماري سیستمیک مانند دیابت، بیماری قلبی، آسم، آرتريت روماتوئيد، واسکولیت، بدخيمي، اپي‌لپسي، نارسایی کلیه یا کبدی.
4. وجود بیماری التهابی یا عفونی حاد
5. حضور حملات سردرد تیپ تنشی در ماه گذشته
6. مصرف مسکن یا داروی اختصاصی میگرن (مانند ارگوتامين) در 8 ساعت گذشته یا مصرف داروي مسكن در 4 ساعت اخیر که ممکن است باعث تسكين درد شود.
7. سابقه آلرژی شناخته شده به سديم والپروات .
8. بیماراني كه برای پروفیلاکسی داروي سديم والپروات خوراكي مصرف مي‌كنند.

پس از توضيح اهداف طرح براي بيماران و اخذ رضايتنامه كتبي از ايشان، بيماران به صورت تصادفي به يكي از دو گروه مداخله و كنترل اختصاص داده خواهند شد. قبل از تجويز دارو، علائم باليني بيماران (نظير تهوع، استفراغ تاری دید، فوتوفوبی و فونوفوبی) و شدت درد بر اساس مقياس VAS اندازه‌گيري و ثبت مي‌شود. سپس بيماران گروه مداخله 400 ميلي‌گرم سديم والپروات را به صورت محلول در 50 سانتي‌متر مكعب نرمال سالين از طريق انفوزيون وريدي در مدت 15 دقيقه به صورت سينگل دوز دريافت خواهند نمود. بيماران گروه كنترل به همان روش ذكر شده براي گروه مداخله، تحت درمان با 8 ميلي‌گرم دگزامتازون به صورت سينگل دوز قرار خواهند گرفت. داروها فاقد اتيكت بوده و نه پزشك و نه بيمار از نوع داروي تجويز شده اطلاعي نخواهند داشت. مجدداً علائم باليني بيماران، شدت درد و همچنين عوارض جانبي احتمالي داروها 30 دقیقه و دو ساعت پس از تجويز دارو مورد بررسي قرار خواهند گرفت.

براي تصادفي نمودن افراد از روش Balance Block Randomization استفاده خواهد. براي اين منظور داروهاي در بلوك‌هاي چهارتايي همانند جدول ذيل قرار خواهند گرفت. سپس خانه‌هاي جدول از يك نقطه به صورت تصادفي از شماره 1 تا 72 كد‌گذاري خواهند شد. هر دارو در جعبه‌اي در بسته كه برچسب كد مربوطه بر روي آن الصاق شده باشد قرار داده خواهد شد به نحويكه پزشك و بيمار هيچ يك از نوع داروي مورد استفاده اطلاعي نخواهند داشت. در پايان مطالعه داروها رمزگشايي شده و بيماران اختصاص يافته به هر گروه مشخص خواهند شد.

D:Dexamethasone

S: Sodium Valproate

| 01_D_ | 02_D_ | 03_S_ | 04_S_ | 05_S_ | 06_S_ | 07_D_ | 08_D_ | 09_D_ | 10_S_ | 11_S_ | 12_D_ | 13_S_ | 14_D_ | 15_D_ | 16_S_ |
| --- | --- | --- | --- | --- | --- | --- | --- | --- | --- | --- | --- | --- | --- | --- | --- |
| 17_D_ | 18_D_ | 19_S_ | 20_S_ | 21_S_ | 22_S_ | 23_D_ | 24_D_ | 25_D_ | 26_S_ | 27_S_ | 28_D_ | 29_S_ | 30_D_ | 31_D_ | 32_S_ |
| 33_D_ | 34_D_ | 35_S_ | 36_S_ | 37_S_ | 38_S_ | 39_D_ | 40_D_ | 41_D_ | 42_S_ | 43_S_ | 44_D_ | 45_S_ | 46_D_ | 47_D_ | 48_S_ |
| 49_D_ | 50_D_ | 51_S_ | 52_S_ | 53_S_ | 54_S_ | 55_D_ | 56_D_ | 57_D_ | 58_S_ | 59_S_ | 60_D_ | 61_S_ | 62_D_ | 63_D_ | 64_S_ |
| 65_D_ | 66_D_ | 67_S_ | 68_S_ | 69_S_ | 70_S_ | 71_D_ | 72_D_ |  |  |  |  |  |  |  |  |

**روش محاسبه حجم نمونه و تعداد آن**

بر اساس نتايج مطالعات پيشين، ميزان بهبودي علائم ميگرن متعاقب تجويز سديم والپروات حدود 73% و دگزامتازون حدود 33% برآورد گرديده است بنابراین حجم نمونه در سطح خطای 5٪ و توان آماری 80٪ به صورت زیر محاسبه گردید.


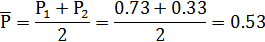


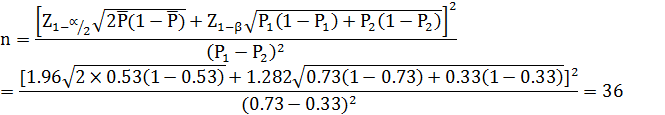


بنابراين حجم نمونه براي هر يك از گروه‌هاي تحت مطالعه برابر 36 نفر و مجموعاً 72 مورد خواهد بود.

**مشخصات ابزار جمع آوري اطلاعات و نحوه جمع آوري آن**

*داده‌هاي مورد نياز از طريق گرفتن شرح حال و انجام معاينه باليني اخذ و در پرسشنامه مربوطه ثبت خواهد شد (ضمیمه شماره 1). پرسشنامه از دو بخش تشكيل شده است. بخش نخست پرسشنامه شامل اطلاعات دموگرافيك (شامل سن و جنسيت) و شرح حال بيمار (شامل تهوع و علائم بينايي) مي‌باشد. بخش دوم پرسشنامه مربوط به بررسي شدت درد مي‌باشد كه به روش* VAS *اندازه‌گيري مي‌شود. بر اين اساس شدت درد از صفر تا 10 درجه‌بندي شده است و از بيمار خواسته خواهد شد تا شدت درد خود را بر اساس اين مقياس تعيين كند. به اين ترتيب به شدت درد از صفر تا 10 نمره داده خواهد شد.*

*براي بررسي و مقايسه داده‌هاي اسمي از آزمون آماري كاي دو و براي داده‌هاي رتبه‌اي از آزمون* Mann-Whitney *استفاده مي‌گردد. همچنين براي بررسي اندازه اثر داروي سديم والپروات نسبت به دگزامتازون از روش آماري رگرسيون لجستيك استفاده مي‌شود. كليه تجزيه و تحليل‌هاي آماري با استفاده از نرم‌افزار* Stata 11 *در سطح اطمينان 95% محاسبه و گزارش خواهد شد.*

**ملاحظات اخلاقي و فرم رضايتنامه مربوطه**

قبل از انجام تحقيق، با تك تك بيماران راجع به اهداف طرح مصاحبه خواهد شد. در صورت تمايل از ايشان رضايتنامه كتبي اخذ خواهد شد (ضمیمه شماره 2). افرادي كه وارد مطالعه شوند هيچگونه هزينه‌اي بابت دريافت دارو و معاينات صورت گرفته پرداخت نخواهند نمود و در هر زماني كه بخواهند مي‌توانند آزادانه و بي هيچ محدوديتي از مطالعه خارج شوند. مجريان وظيفه دارند كه در صورت بروز هرگونه عارضه جانبي احتمالي ناشي از تجويز دارو، نسبت به رفع آن تلاش نمايند.

**محدوديتهاي اجرايي طرح و روش كاهش آنها**

با توجه به اينكه از كليه بيماران رضايتنامه كتبي اخذ مي‌گردد ممكن است اين كار بيماران را دچار واهمه نموده و از پذيرش مطالعه خودداري نمايند. وليكن پيش‌بيني مي‌شود كه ارائه توضيحات كافي و توجيه آنها بتوان رضايت ايشان را براي ورود به مطالعه جلب نمود.

جدول متغيرها

| متغير | مستقل | وابسته | كمي | | كيفي | | تعريف | نحوه اندازه گيري | مقياس |
| --- | --- | --- | --- | --- | --- | --- | --- | --- | --- |
|  |  |  | پيوسته | گسسته | اسمي | رتبه‌اي |  |  |  |
| سن |  |  |  |  |  |  | تعداد سال‌های سپري شده از عمر فرد | پرسش از آزمودني | سال شمسي |
| جنس |  |  |  |  |  |  | فنوتیپ بیمار برحسب زن ومرد | مشاهده آزمونی | مذكر/ مؤنث |
| درمان |  |  |  |  |  |  | داروي تزريقي از گروه كورتيكواستروئيدها/ داروی تزریقی ضدتشنج مهار کننده کانال سدیم و GABA | ميلي‌گرم | دگزامتازون/ سدیم والپروات |
| پاسخ به درمان (شدت درد) |  |  |  |  |  |  | شدت درد بر اساس مقياس VAS | پرسشنامه | صفر تا 10 |
| تهوع |  |  |  |  |  |  | احساس ناخوشايندي كه قبل از استفراغ به بيمار دست مي‌دهد. | پرسش از آزمودني | دارد/ ندارد |
| علائم بينايي |  |  |  |  |  |  | مشاهده جرقه‌هاي نوراني در ميدان بيانيي به صورت گذرا | پرسش از آزمودني | دارد/ ندارد |

**جدول زمان بندي مراحل اجراي طرح**

| رديف | فعاليت‌هاي اجرايي | زمان كل | زمان اجرا به ماه | | | | | | | | | |
| --- | --- | --- | --- | --- | --- | --- | --- | --- | --- | --- | --- | --- |
|  |  |  | 1 | 2 | 3 | 4 | 5 | 6 | 7 | 8 | 9 | 10 |
|  | ثبت پروپوزال |  |  |  |  |  |  |  |  |  |  |  |
|  | جمع آوري نمونه |  |  |  |  |  |  |  |  |  |  |  |
|  | انجام آزمايشات |  |  |  |  |  |  |  |  |  |  |  |
|  | ورود اطلاعات به رايانه |  |  |  |  |  |  |  |  |  |  |  |
|  | آناليز |  |  |  |  |  |  |  |  |  |  |  |
|  | تهيه گزارش نهايي |  |  |  |  |  |  |  |  |  |  |  |

**منابع علمی**

1. Chang M, Rapoport AM. Acute treatment of migraine, headache. Techniques in regional Anesthesia & Pain Management. 2009?;13:9-15.

2. Hu XH, Markson LE, Lipton RB. Burden of migraine in the United States: disability and economic costs. Arch Intern Med. 1999;159:813-8.

3. Desmeules JA. The tramadol option. Eur] Pain. 2000;4(SuppI A):15-21.

4. KIo U. Tramadol-the impact of its pharmacokinetic and pharmacodynamic properties on the clinical management of pain. Arzneimittelforschung. 2003;53:681-7.

5. Mattia C, Coluzzi F. Tramadol: Focus on musculoskeletal and neuropathic pain. Minerva Anestesiol. 2005;71:565-84.

6. Silberstein SD, Freitag FG, Rozen TD, al e. Tramadol/acetaminophen for the treatment of acute migraine pain: findings of a randomized, placebocontrolled trial. Headache. 2005;45:1317-27.

7. Headache Classification Committee of the International Headache Society. Classification and diagnostic criteria for headache disorders, cranial neuralgia, and facial pain. Cephalalgia. 1988?;8:1-96.

8. Headache Classification Subcommittee of the International Headache Society. The international classification of headache disorders. 2nd edition Cephalalgia. 2004?;24(Suppl 1):1-151.

9. Gray RN, Goslin RE, McCrory DC, Eberlein K, Tulsky J, Hasselblad V. Drug treatments for the prevention of migraine: technical review 2.3: Agency for Health Care Policy and Research; 1999?

10. Institute for Clinical Systems Improvement. Diagnosis and treatment of headache. Bloomington: Institute for Clinical Systems Improvement; 2004?

11. Silberstein SD. Practice parameter: evidence-based guidelines for migraine headache (an evidence-based review): report of the Quality Standards Subcommittee of the American Academy of Neurology. Neurology. 2000;55(754-762).

12. Bahra A, Matharu MS, Buchel C, Frackowiak RSJ, Goadsby PJ. Brainstem activation specific to migraine headache. Lancet. 2001;357:1016-7.

13. Menken M, Munsat TL, Toole JF. The global burden of disease study: implications for neurology. Arch Neurol. 2000;57:418-20.

14. Pappagallo M. Newer antiepileptic drugs: possible uses in the treatment of neuropathic pain and migraine. Clin Ther. 2003;25:2506-38.

15. Cutrer FM, Limmroth V, Moskowitz MA. Possible mechanisms of valproate in migraine prophylaxis. Cephalalgia. 1997;17:93-100.

16. Ramadan NM. Current trends in migraine prophylaxis. Headache. 2007;Suppl 1:S52-S7.

17. Bowyer SM, Mason KM, Moran JE, Tepley N, Mitsias PD. Cortical hyperexcitability in migraine patients before and after sodium valproate treatment. J Clin Neurophysiol. 2005;22:65-7.

18. Rogawski MA, Loscher W. The neurobiology of antiepileptic drugs for the treatment of nonepileptic conditions. Nat Med. 2004;10:685-92.

19. Nishikawa T, Scatton B. Inhibitory influence of GABA on central serotonergic transmission. Raphe nuclei as the neuroanatomical site of GABAergic inhibition of cerebral serotonergic neurons. Brain Res. 1985;331:91-103.

20. Cutrer FM, Limmroth V, Ayata G, Moskowitz MA. Valproate reduces c-fos expression in trigeminal nucleus caudalis (TNC) after noxious meningeal stimulation. Cephalalgia 1995;15(suppl 14):96. 1995;15(Suppl 14):96.

21. Frazee LA, Foraker KC. Use of intravenous valproic acid for acute migraine. Ann Pharmacother. 2008;42:403-7.

22. Shahien R, Saleh SA, Bowirrat A. Intravenous sodium valproate aborts =SPAN class=highlight>migraine headaches rapidly. Acta Neurol Scand. 2011;123:257-65.

23. Stilman MJ, Zajac D, Rybicki LA. Treatment of primary headache disorders with intravenous valproate: initial outpatient experience. Headache. 2004;44:65-9.

24. Mathew NT, Kailasam J, Meadors L, Chernyschev O, Gentry P. Intravenous valproate sodium (depacon) aborts migraine rapidly: a preliminary report. Headache. 2000;40:720-3.

25. Tanen DA, Miller S, French T, Riffenburgh RH. Intravenous sodium valproate versus prochlorperazine for the emergency department treatment of acute migraine headaches: A prospective, randomized, double-blind trial. Ann Emerg Med. 2003;41:847-53.

26. Aronson MD. Nonsteroidal anti-inflammatory drugs, traditional opioids, and tramadol: Contrasting therapies for the treatment of chronic pain. Clin Ther. 1997;19:420-32.

27. Avraham SB, Har-Gil M, Watemberg N. Acute confusional migraine in an adolescent: response to intravenous valproate. Pediatrics. 2010;125:e956–e9.

28. Bigal M, Sheftell F, Tepper S, Tepper D, Ho TW, Rapoport A. A randomized double-blind study comparing rizatriptan, dexamethasone, and the combination of both in the acute treatment of menstrually related migraine. Headache. 2008;48(9):1286-93.

## قسمت سوم – اطلاعات مربوط به هزينه‏ها

**هزينه كارمندي (پرسنلي) باذكر مشخصات كامل و ميزان اشتغال هرفرد و حق الزحمه آنها** :

| ردیف | نوع فعاليت | نام فرد يا افراد | رتبه علمي | تعداد ساعات | حق الزحمه براي يك نفردر هر ساعت | جمع كل |
| --- | --- | --- | --- | --- | --- | --- |
| 1 | تهيه پروپوزال | دکتر مظاهری  دکتر پورالعجل | استادیار  استادیار | 30  20 | 100000  150000 | 3000000  3000000 |
| 2 | جمع آوري اطلاعات | دونفر همکار | کارشناس | 100 | 25000 | 2500000 |
| 3 | استخراج | دونفر همکار | پزشک عمومی | 100 | 35000 | 3500000 |
| 4 | تجزيه و تحليل | دکتر پورالعجل | استادیار | 20 | 150000 | 3000000 |
| 5 | تهيه گزارش نهائي | دکتر مظاهری | استادیار | 35 | 100000 | 3500000 |
| جمع هزينه | |  |  |  |  | 18500000 |

**هزينه آزمايشها وخدمات تخصصي كه توسط دانشگاه ويا ديگر موسسات صورت مي گيرد**:

| جمع ( ريال ) | هزينه براي هر دفعه آزمايش | تعداد كل دفعات آزمايش | مركزسرويس دهنده | موضوع آزمايش يا خدمات تخصصي |
| --- | --- | --- | --- | --- |
|  |  |  |  |  |
| جمع هزينه هاي آزمايشها : | | | | |

**فهرست وسايل و موادي كه بايد از اعتبار اين طرح از داخل يا خارج كشور خريداري شود**:

وسايل غيرمصرفي:

| قيمت كل(ريال) | قيمت واحد (ريال) | تعداد یا مقدار لازم | شركت فروشنده ايراني | شركت سازنده | كشورسازنده | نام دستگاه |
| --- | --- | --- | --- | --- | --- | --- |
| **-** | **-** | **-** | **-** | **-** | **-** | **-** |

مواد مصرفي :

| قيمت كل (ريال) | قيمت واحد (ريال) | تعداديامقدار لازم | شركت فروشنده ايران | شركت سازنده | كشور سازنده | نام ماده |
| --- | --- | --- | --- | --- | --- | --- |
| 336000 | 4200 | 80 | داروپخش | کاسپین | ایران | آمپول دگزامتازون |
| 10800000 | 260000 | 40 | داروپخش | sanofi | فرانسه | آمپول سدیم والپروات (دپاکن) |
| 220000 | 550 | 40 عدد 5 سی سی | ربینا بازار | ربینا بازار | ایران | سرنگ |
| 180000 | 4500 | 40 عدد 50 سی‌سی | ربینا بازار | ربینا بازار | ایران | سرنگ |
| 23440 | 293 | 80 | داروپخش | ثامن مشهد | ایران | الکل و پنبه |
| 776000 | 9700 | 80 | داروپخش | ثامن مشهد | ایران | سرم نرمال سالین 500 سی سی |
| 276000 | 3700 | 80 | داروپخش | mediflon | هند | آنژیوکت |
| 12611440 |  |  |  |  |  | جمع هزينه |

هزينه مسافرت :

| هزينه به ريال | تعداد افراد | نوع وسيله نقليه | تعداد مسافرت در مدت اجراي طرح و منظور آن | مقصد |
| --- | --- | --- | --- | --- |
| - | - | - | - | - |
|  | جمع هزينه هاي مسافرت: | | | |

هزينه هاي ديگر

| 500000 ریال | هزينه هاي تكثير اوراق |
| --- | --- |
|  | ساير موارد (هزينه تکرار برخی آزمايشات بر حسب مورد) |
|  | جمع هزينه |

جمع هزينه هاي طرح :

|  | هزينه مسافرت | 18500000 | هزينه پرسنلي |
| --- | --- | --- | --- |
| 500000 | هزينه هاي ديگر |  | هزينه آزمايشها و خدمات تخصصي |
|  | متفرقه | 12611440 | هزينه مواد و وسايل مصرفي |
| 31611440 | جمع كل |  | هزينه وسايل غير مصرفي |

مبلغي كه از منابع ديگر كمك خواهد شد و نحوه مصرف آن : ……………….……………. ريال

باقيمانده هزينه هاي طرح كه تامين آن درخواست مي شود : ……………………..………. ريال

با مطالعه قسمت اول اين فرم و رعايت مفاد آن بدينوسيله صحت مطالب مندرج در پيش نويس را تائيد مينمايد واعلام ميداردكه اين تحقيق صرفاً به صورت :

1. يك طرح تحقيقاتي در دانشگاه علوم پزشكي همدان
2. بصورت مشترك با ……
3. در قالب پايان نامه در رشته ................................... ارائه شده است.

نام و نام خانوادگي مجري يا مجريان طرح يا استاد راهنما

محل امضاء
