# Supplementary material for: Effect of Intravenous Sodium Valproate vs Dexamethasone on Acute Migraine Headache: A Double Blind Randomized Clinical Trial
Source: PLoS One. 2015 Mar 20;10(3):e0120229. doi: 10.1371/journal.pone.0120229 (PMC4368536; doi:10.1371/journal.pone.0120229)
Supplement: S1 Protocol — (DOCX) [file pone.0120229.s002.docx]

**Dear Editor-in-Chief,**

Subject: Supplement A

This supplement contains the following items:

1.	Original protocol and statistical analysis plan

2.	Summary of changes

Sincerely yours,

**Jalal Poorolajal (MD, PhD)**

Associate Professor of Epidemiology, Modeling of Noncommunicable Diseases Research Center, Department of Epidemiology & Biostatistics, School of Public Health, Hamadan University of Medical Sciences, Hamadan, Iran

E-mail 1: poorolajal@umsha.ac.ir

E-mail 2: poorolajal@yahoo.com

**Original protocol and statistical analysis plan**

**The effect of intravenous sodium valproate versus dexamethasone on headache relief in patients with acute migraine headache: a double blinded randomized clinical trial**

**Objectives**: To investigate the effect of intravenous sodium valproate versus dexamethasone on acute migraine headache.

**Design**: Double blinded randomized controlled trial.

**Setting and conduct**: Patients who will refer to emergency ward of Beasat and Farshchian hospitals during study period.

**Participants including major eligibility criteria**: age of 18 to 65 years; history of having migraine for at least one year; severity of headache equal to or greater than 5 VAS.

**Intervention**: A single dose of 400 mg sodium valproate plus 50 mL saline normal solution within 15 min intravenously, versus a single dose of 16 mg dexamethasone plus 50 mL saline normal solution within 15 min intravenously.

**Main outcome measures**: Severity of headache using VAS scale will be measured at baseline, one half and two hours later.

**Gender**: Both male and female

**Age**: 18-65 years

**Sample size**: 72

**Condition ICD-10 code**: G43 Migraine with or without aura

**Ethical approval**: Research Ethic Committee of Hamadan University of Medical Sciences

**Date**: 2012-02-27

**Number**: D/P/16/35/9/4452

**IRCT registration number:** IRCT201202199014N1

**Expected recruitment start date**: 2012-04-21

**Expected recruitment end date**: 2012-10-23

**Statistical analysis plan**

The independent and paired t-tests were used for bivariate analysis and ANOVA test for multivariate analysis of continuous variables and chi-square test for analyzing categorical variables. All statistical analyses were performed at a significance level of 0.05 using Stata software, version 11 (StataCorp, College Station, TX, USA).

**********************************************

**Summary of changes**

**Intervention**: A single dose of 400 mg sodium valproate plus 50 mL saline normal solution within 15 min intravenously, versus a single dose of 16 mg dexamethasone plus 50 mL saline normal solution within 15 min intravenously.
